# Community Knowledge, Attitudes and Practices About Malaria: Insights from a Northwestern Colombian Endemic Locality

**DOI:** 10.3390/tropicalmed9110281

**Published:** 2024-11-18

**Authors:** Paola Muñoz-Laiton, Juan C. Hernández-Valencia, Margarita M. Correa

**Affiliations:** Grupo Microbiología Molecular, Escuela de Microbiología, Universidad de Antioquia, Medellín 050010, Colombia; paola.munoz1@udea.edu.co (P.M.-L.); juan.hernandez21@udea.edu.co (J.C.H.-V.)

**Keywords:** KAP, knowledge, attitudes, practices, malaria, Colombia

## Abstract

Malaria prevention and control programs are mainly oriented to vector control, timely diagnosis and adequate treatment. Malaria transmission is influenced by several factors, including biological and social aspects. Thus, it is relevant to consider community beliefs and practices to ensure sustainable prevention and control strategies. This study aimed to determine knowledge, attitudes and practices (KAP) towards malaria in an endemic locality in northwestern Colombia. Preliminary data were collected through a focus group discussion. Subsequently, a KAP survey was administered to the community. KAP scores were associated with both sociodemographic characteristics and with previous malaria infection. Focus group data revealed knowledge gaps and the absence of or having worn-out nets. Survey results showed that participants recognized a mosquito bite as the transmission mode (72.09%), followed by dirty water (44.19%), high fever (86.05%) and headache (79.07%) as the main symptoms. Regarding attitudes, 44.19% of the people would go to the hospital in the case of having symptoms. The most recognized practices for disease prevention were the use of mosquito nets (65.12%) and fans (23.26%). The results showed that some people had misconceptions about the disease transmission mode. The analysis showed significant associations of either female gender and homemaker occupation with a good knowledge [OR = 3.74, (*p* = 0.04), OR = 3.55, (*p* = 0.04), respectively] or female with a positive attitude towards malaria control and prevention [OR = 4.80, (*p* = 0.04)]. These results showed that the identified gaps in KAP require increasing education among the community in addition to applying public health prevention efforts. The data may be useful in designing malaria control strategies that involve community participation.

## 1. Introduction

Colombia is the third most malaria-burdened country in the American continent [1]. In 2023, a total of 102,455 cases were reported in Colombia; this corresponds to an increase in the data from 2022 (71,573 cases) and 2021 (72,022 cases) [2,3,4]. Historically, the most malaria-endemic areas of the country have been the Pacific region in the west and the Urabá-Bajo Cauca-Alto Sinú region in the northwest [5]. Specifically, the Bajo Cauca subregion, where this work was conducted, represents an important transmission focus [6,7]. Currently, malaria control and prevention programs are mainly focused on decreasing transmission through *Anopheles* mosquito control, using long-lasting insecticide-treated nets (LLINs) and indoor residual spraying (IRS), and through timely diagnosis and treatment. However, these programs do not consider the participation and knowledge of the communities at risk [7,8]. Considering that malaria is a relevant problem to public health, which encompasses biological and social components, research aimed to define actions for disease mitigation must be articulated with the social dynamics of the communities. Therefore, knowledge gaps in the communities regarding malaria should be considered to promote and reinforce appropriate knowledge and practices for disease prevention and control. It will ensure achieving effective and sustainable results [9,10].

The Global Technical Strategy for Malaria 2016–2030 reinforces the need to include the community to encourage their participation in malaria control efforts [11]; however, individual willingness to participate in the strategies is influenced by personal knowledge and attitudes toward a disease [12]. In this sense, beliefs and attitudes towards malaria can influence behaviors that may either increase the risk of contracting the disease or, on the contrary, reinforce protective measures. For example, the non-use of mosquito nets or their misuse as fishing nets was associated with their misunderstanding about the importance of using them, as well as the belief that they will become ill and have various adverse symptoms by sleeping under nets [12,13,14].

Focus group discussions and surveys on knowledge, attitudes and practices (KAP) are relevant for gathering crucial information for the design of focused educational programs and for ensuring community participation, acceptance and adherence to control and prevention strategies [15,16,17]. The few studies conducted in Colombia to investigate social aspects related to malaria indicate that disease risk is associated with sociodemographic factors, such as the number of people per household, occupation, educational level and family income [18,19]. In general, community perceptions of the disease are not considered [20,21]. Studies applying malaria KAP surveys have been conducted in the Colombian Pacific region [19,21,22,23,24] and in the Alto Sinú subregion [22]; however, there are no data regarding this type of study from the Bajo Cauca subregion. In the fight against malaria, it is crucial to understand community perceptions, which are mainly influenced by the local or specific context. Considering this, this work aimed to determine the community’s knowledge, attitudes and practices towards this disease in an endemic locality of Bajo Cauca. In addition, determinants associated with KAP related to malaria were characterized.

## 2. Materials and Methods

### 2.1. Study Design and Area

**Figure 1:**
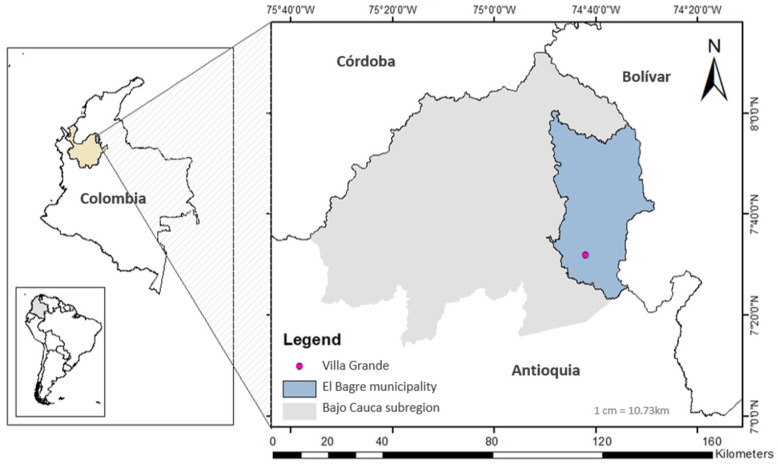
Study area. The left square shows the map of Colombia in relation to South America and the Bajo Cauca subregion in northwestern Colombia. The right square shows the Villa Grande locality in the BC subregion.

A descriptive, cross-sectional study was conducted in the Villa Grande locality (7.5410 Latitude, −74.7025 Longitude), El Bagre municipality, situated in the malaria-endemic Bajo Cauca-BC subregion, Colombia (Figure 1), from June to August 2021. Historically, this municipality has contributed to the large number of malaria cases in the BC subregion [6,25]. Malaria transmission is constant throughout the year [26]. The nearest diagnostic post is 30 min away by motorbike, but to access treatment, people must often travel to the municipal seat, located approximately one and a half hours away by motorbike. Diagnosis is mainly performed by thick smear, and the predominant circulating parasite is by *Plasmodium vivax* [6,27]. The main malaria vectors in the municipality are *Anopheles darlingi* and *Anopheles nuneztovari* [28,29]. According to a local census from 2018, Villa Grande harbors 37 families and approximately 75 people; 24 of them are children.

### 2.2. Focus Group Discussion

Initially, a focus group discussion was conducted with 11 people from the locality to explore community knowledge of malaria and their practices towards its prevention. The information obtained served to revise and improve the questionnaire for the KAP survey. Participants included community leaders and individuals randomly selected from the community by local facilitators. The focus group dialogue was moderated by a member of the research team who formulated questions to facilitate the discussion during a session that lasted approximately 1 h. The dialogue was recorded after receiving oral consent from the participants. Subsequently, the narrative was transcribed to perform a qualitative analysis that allowed information grouping into categories and subcategories. The category “knowledge about malaria” encompassed the subcategories “cause and transmission of malaria” and “symptoms”. The category “attitudes and practices towards malaria” was divided into “attitudes” and “practices”. The category “problems related to malaria and community participation” encompassed the subcategories “impact of malaria in the community” and “community expectations and proposals”.

### 2.3. Knowledge, Attitudes and Practices Survey and Data Analysis

The KAP survey was designed and modified based on a previous study by the World Health Organization and the Pan American Health Organization [30]. The survey contained 42 questions distributed into four thematic areas: (i) sociodemographic characteristics, (ii) knowledge about malaria (e.g., transmission mechanism, symptoms and diagnosis), (iii) attitudes (e.g., search for malaria treatment) and (iv) malaria prevention practices (e.g., mosquito net use and adoption of additional protective measures). Before administration, the KAP survey was reviewed by an interdisciplinary team to ensure that the questions were understandable and covered the necessary information and to guarantee its comprehension and adequacy to the local dialect. To select participants for the KAP survey, a convenience, non-probability sampling was conducted. The sample size was calculated using OpenEpi, v. 3.01 [31] with a 70% expected frequency, 5% acceptable margin of error and 1.0% design effect. A sample size of 45 was estimated with a 95% confidence level. Surveys were paper-based, and data were collected house-to-house by interviews performed by trained field staff in Spanish. Participants in the focus group also completed the survey as the information from each tool enabled different analyses, and both included different questions.

The inclusion criteria for participating in the KAP survey included being of legal age (≥18 years) and agreement to sign the informed consent. The research protocol was approved by a Bioethics Committee of Universidad de Antioquia, Colombia (CBE-SIU, 2241977).

Survey data were recorded in Epi Info ^TM^, v. 7.2.4.0 [32]. Two people from the research team reviewed the data independently to detect possible errors included during registration. Results of the KAP survey were quantitatively analyzed using descriptive statistics, including frequency distribution and percentages. To determine the score of the participant’s knowledge, attitudes and practices, each correct answer was scored with 1, while an incorrect or no answer was 0. A modified Bloom’s cut-off was applied, and a score > 60% was considered “Good” or “Positive”, and below 60%, “Poor” or “Negative” [33,34]. In addition, a bivariate analysis was conducted to assess the relationships between KAP scores with relevant sociodemographic characteristics and a previous malaria infection. Data were analyzed in 2 × 2 contingency tables, and the odds ratio (OR) was calculated. Age was classified into two groups, considering a proportional distribution. A *p*-value of 0.05 or less was considered significant.

## 3. Results

### 3.1. Results of the Focus Group Discussion

**Figure 2:**
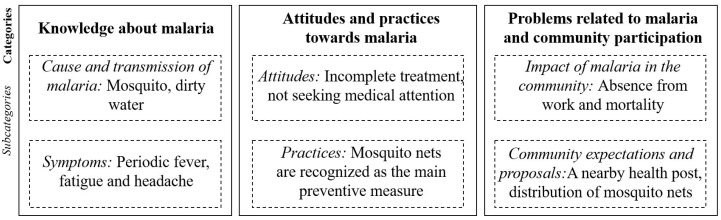
Diagram of categories and subcategories established after qualitative analysis of the focus group discussion.

A qualitative analysis allowed the grouping of the responses obtained during the focus group discussion into categories and subcategories (Figure 2). Regarding the knowledge about malaria, people believed that the disease was transmitted by a mosquito or dirty water. In relation to the “symptoms” subcategory, participants identified periodic fever, fatigue and headache as the main symptoms of malaria. The community referred to the disease as “paludismo”, “paludismo vivax” and “paludismo falciparum”, and they recognized that symptoms of each type differed. Although “malaria” was not the term commonly used to name the disease, they called the workers from the health services that carried out activities of malaria control and prevention “malarios”.

Regarding “attitudes towards malaria”, some unfavorable behaviors were identified. For instance, having incomplete treatments, which may increase disease risk; some individuals mentioned: “I should have taken three pills and took two”. Another negative attitude is not seeking appropriate medical attention in case of illness. Instead, people often consulted private healthcare workers or traditional healers (traditional medicine) due to the long waiting times at health services (western medicine) and their lack of trust in medical practitioners. Even more, this study spanned the time of the COVID-19 pandemic, which made this attitude more evident. For instance, some individuals expressed their fears of going out and preferred getting malaria over contracting the virus: “people are afraid to go out, they prefer to be killed by malaria”. Regarding the “practices” subcategory, it is noteworthy that the community recognized the use of mosquito nets as the main preventive measure, not only against mosquitoes but against a variety of wild animals such as snakes, spiders and even felines. In addition, a focus group participant stressed the importance of using bed nets since mosquitoes usually bite at night.

In terms of the “impact of malaria in the community”, people recognized malaria as a community problem because it causes absence from work and mortality. Related to the “community expectations and proposals” subcategory, participants who had more knowledge about the mosquito life cycle mentioned that they should organize themselves to clean ditches and water tanks. Additionally, they identified the need for a health post close to the locality to provide malaria diagnosis and treatment; this will prevent them from traveling long distances to seek diagnosis in other villages or the municipality. In addition, participants highlighted the need for mosquito net provision; many of them did not have one, or it was worn out.

### 3.2. Knowledge, Attitudes and Practices-KAP Survey

#### 3.2.1. Sociodemographic Characteristics

The KAP survey was administered to 43 people in 33 houses of Villa Grande. The majority of people were women (62.79%). Participants’ average age was 43 ± 16.1 years old. The oldest person was a 79-year-old man. Participants have lived in the locality on average for 14 ± 15.4 years (Me = 9, IQR, interquartile range = 22). In the month before the survey, the people’s mobility outside the locality was 46.51% (IC 95% = 31.18–62.35), mainly traveling to the El Bagre urban area (75.00%); the main reasons were visiting relatives (35.00%), health care seeking (10.00%) and working (10.00%). Regarding education, 41.86% of the surveyed finished high school and 32.56% primary school. Most of the participants lived in unmarried cohabitation (53.49%). Almost all of them had subsidized health affiliation (86.05%), which relates to the health system category for poor populations to have access to health services through funding by the State. The main occupation was homemaker, followed by others (apiculture, mechanics and transportation) (Table 1). The average number of inhabitants per house was 3.72 ± 2.08 (Me = 3.7, IQR = 3)

Regarding dwellings, all people had a house to live in; some of them were owners (78.13%), and others lived in borrowed houses assigned by community leaders. The predominant material was an aluminum–zinc roof, walls made of wooden board and earthen floors (Table 2). Regarding basic services, 93.94% of the houses had public electricity (CI 95% = 79.77–99.26) and 100% had a water supply through a river, stream or rainwater collection tank (95% CI = 89.12–100); however, none of the homes had access to drinking water. A total of 60.61% had a latrine (CI 95% = 42.14–77.09) and 78.79% cooked with a gas tank (CI 95% = 61.09–91.02). The houses lacked water pipelines, sewage systems and a garbage collection scheme.

During the evening hours, between 6:00 and 10:00 p.m., most people preferred spending their leisure time outside the house in open and unwalled spaces (58.14%; CI 95% = 27.01–57.87). However, some people chose to stay inside the house (39.53%; CI 95% = 24.98–55.59) or go to nearby villages (15–30 min by motorbike) to engage in leisure activities. Among the leisure activities performed during this time were watching television or talking with neighbors.

#### 3.2.2. Malaria Knowledge

In general, the community named the disease “paludismo” (55.81%). However, 34.38% of the people used the term “paludismo falciparum” or 30.23% used the term “paludismo vivax”; these responses resemble those from the focus group. The disease was recognized as a problem (86.04%), especially because it causes absence from work. Most participants acknowledged disease prevention as an individual responsibility (51.16%). In addition, 72.09% recognized that its transmission occurs by a mosquito bite, followed by dirty water (44.19%). It is noteworthy that 30.23% of the participants mentioned that transmission occurs through both mosquito bites and dirty water. In general, participants had adequate knowledge of the symptoms, with high and periodic fever, headache and chills being the main symptoms (Table 3). Of note, more than half of the participants acknowledged having malaria while living in Villa Grande (55.81%, CI 95% = 39.88–70.92).

#### 3.2.3. Attitude Towards Malaria Control and Prevention

More than 90% of the people agreed with the importance of having a full malaria treatment and indoor residual spraying. In general, a large part of participants considered that health care assistance is useful for recovering (44.19%), followed by taking traditional medicine (25.58%) (Table 4). Plants frequently used by the community to treat malaria were “quina” (*Cinchona officinalis*), “balsamina” (*Momordica balsamina*), “cedrón” (*Aloysia citrodora*), “gualanday” (*Jacaranda mimosifolia*) and bitter plants like “contragavilana” (*Neurolaena lobata*). Scientific names in parentheses are the probable taxonomic classifications of the common names given by people.

#### 3.2.4. Practices for Malaria Prevention

The prevention measure most recognized by the community is a mosquito net (65.12%), followed by the use of a fan (23.26%) (Table 5). Of the 33 dwellings, 9 did not have any mosquito net (27.27%; CI 95% = 13.30–45.52), 12 had one (36.36%; CI 95% = 20.40–54.88) and 12 had two or more (36.36%; CI 95% = 20.40–54.88). In more than half of the homes, all inhabitants used a mosquito net to sleep (54.17%); however, frequently, these nets were very worn out (P. Muñoz, personal observation). The hours of mosquito net use were mainly between 9:00 p.m. (34.78%; CI 95% = 16.38–57.27) and 6:00 a.m. (39.13%; CI 95% = 19.71–61.46). Of note, in one house, the mosquito net was used for purposes other than to prevent malaria; it was torn, and parts were used to cover a water tank or a hen cage.

### 3.3. Malaria and COVID-19

The KAP survey included a section of questions related to COVID-19. Of the total participants, 39.53% stated they had COVID-19 symptoms (headache, lack of appetite and fever). One woman was reported to be seeking attention at the hospital and not being tested for malaria. In the case of symptoms, 51.16% of participants would not attend the hospital; they preferred to go to traditional healers for diagnosis and treatment. Most of the participants considered that the symptoms of malaria and COVID-19 were similar (53.49%), while 25.58% mentioned that they were different and 20.93% did not know.

### 3.4. Relationship Between Focus Group Discussion and KAP Survey

In general, the responses from people in the focus group discussion and during the KAP survey agreed regarding knowledge of the disease (transmission and symptoms), attitudes and practices, except for the response related to the malaria treatment. In the KAP survey, 44.19% of people indicated that they would attend the hospital if they had symptoms. Conversely, during the focus group discussion, all participants stated that they would not attend the hospital for fear of getting COVID-19, opting for visiting a traditional healer.

### 3.5. KAP Scores and Association with Sociodemographic Characteristics and with Previous Malaria Infection

According to the results of the knowledge score assessment, 51.16% had good knowledge about malaria and 79.07% had a positive attitude against its prevention. For the score on practices for malaria prevention, 4.65% of participants had a score of > 60%, and 53.50% of the participants had a score of ≥ 40% (Table S1). Hence, participants recognized mosquito nets as a main preventive measure, but the practice score indicated that they did not use additional protection practices like repellent or fumigation.

In the bivariate analysis, an association of KAP with sociodemographic factors evidenced that gender and occupation were significantly associated with a good malaria knowledge score. Female participants [OR = 3.74, (*p* = 0.04)] and homemakers [OR = 3.55, (*p* = 0.04)] had better knowledge of the disease (Table 6). Additionally, being female was associated with a positive attitude [OR = 4.80, (*p* = 0.04)] (Table 7). Age, education or previous malaria infection were not significantly associated with a good knowledge score or a positive attitude. None of the evaluated sociodemographic characteristics or previous malaria episodes were associated with the practice score (Table S2). 

## 4. Discussion

This study, conducted to determine knowledge, attitudes and practices related to malaria in a Colombian endemic locality, provided essential information on social aspects of the disease. Analysis of the sociodemographic characteristics revealed a larger participation of women, attributed to their involvement in home activities, while men were engaged in specific economic activities out of their homes (e.g., mining, agriculture, etc.). Of note, the female-male ratio in this community was 1:1. Regarding the educational level of the community, it was low; 23.26% of the participants were illiterate and 32.56% only had partial or complete primary education. A limited educational level has previously been linked to a higher malaria prevalence, and it is also a risk factor for malaria in children [35,36]. Public services were very deficient or completely absent, and homes did not have potable water or sanitation services. These social determinants are suggested to increase malaria risk, as previously observed in Latin America and Africa [18,37]. Concerning house structures, aluminum–zinc roofs with wooden board walls and earthen floors were the most prevalent material types. It is known that house structure affects malaria transmission [38,39]. Houses with wooden board walls were related to higher malaria parasite prevalence than dwellings with cement or brick [40]. Likewise, houses with earth floors were associated with a higher malaria incidence in children from Uganda [41]. The above suggests that the house structure in Villa Grande may provide easy access for mosquitos through spaces between wooden boards, which could increase human–vector contact.

Regarding community knowledge about malaria, most survey participants recognized malaria is transmitted through a mosquito bite. However, some also indicated other sources of transmission; close to half of them associated transmission with dirty water or did not provide any answer. Similar findings were previously reported in studies conducted in the Colombian Pacific region. In those studies, a similar number of participants associated the disease with a mosquito bite, and contrary to the present study, a very low number linked it to water [19,21,22]. Of note, some people recognized both mechanisms, mosquitoes and dirty water, as modes of transmission. This suggests that when implementing educational strategies in this community, it is essential to correct this knowledge gap. The community named the disease “paludismo vivax” or “paludismo falciparum”; this could be attributed to the test result that indicates the parasite species when they were tested. Furthermore, >67% of participants identified the most common symptoms as high and periodic fever, headache and chills. The probable reason for good knowledge about the symptoms is that approximately 55% of the participants suffered from malaria at some point in their lives. In similar studies in the Colombian Pacific, > 69% of the participants had adequate knowledge about the main symptoms [21,23].

According to the analysis of attitudes towards malaria, 95% of the participants have taken or would take treatment for the disease, and a similar number agreed with insecticide spraying to control the spread of malaria. These results are similar to those in a study conducted with the Guna Indigenous population of Panamá, where 85% of people mentioned taking medication and 100% agreed with insecticide spraying in homes [42]. Despite a positive attitude towards medication, the focus group discussion showed that some people did not take the full treatment. This demonstrates the need to reinforce this behavior in the community to avoid relapses caused by *Plasmodium vivax* [43], avoid *Plasmodium* drug resistance [44] and disease evolution towards complicated malaria by *Plasmodium falciparum* [45]. The survey also showed that < 50% of participants would attend a hospital for diagnosis and treatment if they experienced symptoms of malaria. Some of them preferred to use traditional medicine due to mistrust in the healthcare system (western medicine), an attitude exacerbated by the COVID-19 pandemic. Globally, this pandemic had a general negative impact on many diseases, particularly malaria [46,47,48]. Thus, control programs should encourage people to seek healthcare attention for suspected malaria, and health authorities should work on improving trust in healthcare systems. Previous studies have attributed the failure to seek timely treatment in rural areas to aspects such as long distance to health care centers, service quality and waiting times [23]. These reasons were also mentioned by participants in the focus group. To reinforce a positive attitude towards seeking treatment, it is necessary to establish a nearby health facility for timely diagnosis and treatment. Other limitations related to seeking health care were the cost of transportation and other indirect costs; this was previously evidenced among people living in rural areas who avoided seeking treatment and ultimately developed complications from the disease [49].

Regarding practices to prevent malaria, the control measure most recognized by the community was the mosquito net (65.12%); however, many of the nets were in poor condition and did not effectively protect against mosquito bites [50]. In agreement with this observation, a previous study demonstrated an association between “moderately torn” or “too torn” mosquito nets and a higher malaria prevalence [51]. Furthermore, results indicated that mosquito nets were insufficient to protect all people at risk. About 36% of households only had one mosquito net, and on average, there were four people per house. Moreover, most mosquito nets were acquired 2–3 years prior, a period that decreases the effectiveness of LLINs (long-lasting insecticide-treated nets) [52]. This fact suggests the need to provide LLINs for better protection against malaria, and their distribution should be accompanied by educational campaigns to motivate their appropriate use because misuse is evidenced in the community. Prevention measures vary by region, with cultural issues or control programs that may influence locally conducted practices. For instance, the prevention measure most used by the Guna Indigenous population in Panamá is smoking pipes (78%), a cultural behavior, but only 6% use a mosquito net [42]. Conversely, in the Colombian Pacific region, similar studies showed a 50% to 90% mosquito net use [21,22,23]. The second most used prevention measure recognized by the community was the use of a fan (23.26%). In a previous study conducted in experimental huts, among the prevention measures, the use of fans decreased the blood-fed *Anopheles* mosquitoes captured by 27% [53]. Another prevention measure used by the community was burning materials to generate smoke and prevent mosquito bites; however, this practice is not appropriate while people sleep, which is the time when *Anopheles* vectors display higher human biting activity [54,55]. The former indicates the importance of carrying out studies to evaluate the protection measures used by communities and their efficacy in reducing malaria risk.

Regarding the association of KAP with either sociodemographic factors or previous malaria infection, a significant association was found between women, good knowledge and a positive attitude. Moreover, homemaker was associated with good knowledge; this is mainly the occupation of women in the community. Likewise, other studies have indicated the main role of women in providing health care and support to other household members [56]; this makes them more familiar with disease processes. To assess perceptions and practices towards malaria, as related to gender and specifically to women, it is necessary to understand their role in the community and the interplay of sociocultural factors [54,57]. Although approximately half of the population had good knowledge of the disease (51%) and positive attitudes (80%), prevention practices had a low score. It is known that knowledge and positive attitudes towards malaria influence good prevention practices. However, other factors have to be considered, such as the availability of mosquito nets and repellent distribution, in addition to reinforcing drivers that motivate appropriate behaviors, like the involvement of leaders and the community in educational strategies for malaria prevention [58]. Despite the valuable findings and the application of useful strategies to assess baseline information on knowledge, attitudes and practices about malaria, there are limitations to consider and are related to the cross-sectional study, which provides specific information at a single point in time.

## 5. Conclusions

The results of this study allowed the establishment of a baseline on the knowledge, attitudes and practices in a community from a northwestern Colombian endemic locality. In general, people recognize malaria symptoms, but there are misconceptions regarding its transmission. Although most of the community had a positive attitude, the search for healthcare assistance must be reinforced. It was evidenced that the population was skeptical about the healthcare system and attending the hospital, even more so during the COVID-19 pandemic. The female gender was a variable associated with better knowledge and attitudes regarding malaria. The use of preventive measures needs to be strengthened in the community. Educational interventions could improve gaps in the mode of disease transmission, emphasize the importance of seeking healthcare assistance in case of symptoms, and motivate the appropriate use of preventive tools upon distribution of mosquito nets. Altogether, these data could be used in the design and application of effective malaria control strategies.

## Figures and Tables

**Table 1:** Sociodemographic characteristics of the participants from Villa Grande, El Bagre.

Variable		Participants (*n* = 43)	%
Socioeconomic level	Stratification 1 *	43	100
Gender	Female	27	62.79
	Male	16	37.21
Age (years)	18–19	1	2.50
	20–29	12	30.00
	30–39	3	7.50
	40–49	12	30.00
	50–59	4	10.00
	60–69	5	12.50
	70–79	3	7.50
Marital status	Cohabitation	23	53.49
	Single	10	23.26
	Married	9	20.93
	Widow/er	1	2.33
Educational level	Secondary ^§^	18	41.86
	Primary ^§^	14	32.56
	Illiterate	10	23.26
	Technical	1	2.33
Health affiliation	Subsidized	37	86.05
	Contributing	6	13.95
Occupation	Homemaker	25	58.14
	Other	9	20.93
	Agriculture	6	13.95
	Mining	3	6.98

* Corresponds to a government socioeconomic classification where stratification 1 is “low-low” and implies that immovables classified in this category have significant deficiencies in the quality of infrastructure and public services. ^§^ Partial or complete education.

**Table 2:** Housing characteristics in Villa Grande, El Bagre.

Variable		Houses (*n* = 33)	%
Home ownership	Own	25	78.13
	Borrowed	7	21.88
Roof type	Aluminum–zinc tiles	20	60.61
	Shading mesh	5	15.15
	Thatched	4	12.12
	Clay roof tiles	2	6.06
	Plastic bags	1	3.03
	Other	1	3.03
Wall type	Wooden board	29	87.88
	Cement block	4	12.12
Floor type	Earthen	25	75.76
	Cement	8	24.24

**Table 3:** Malaria knowledge of the participants from Villa Grande, El Bagre.

Variable		Participants (*n* = 43)	%
Do you consider malaria a problem?	Yes	37	86.04%
	No	5	11.62%
	Do not know/No answer	1	2.32%
Who is responsible for preventing malaria? *	Each person	22	51.16%
	Health Secretary	19	44.19%
	Community	2	4.65%
	Family	1	2.33%
	Do not know/No answer	3	6.98%
How is malaria transmitted? *	Bite of any mosquito	31	72.09%
	Water	19	44.19%
	Parasite transmitted by mosquito bite	2	4.65%
	Air	1	2.33%
	Bite of *Anopheles* mosquito	1	2.33%
	Do not know/No answer	6	13.95%
	Other	1	2.33%
What are the symptoms of malaria? *	High and periodic fever	37	86.05%
	Headache	34	79.07%
	Chill	29	67.44%
	Muscle and bone pain	15	34.88%
	Vomiting and diarrhea	13	30.23%
	Weakness and tiredness	8	18.60%
	Other	8	18.60%
How have you been diagnosed with malaria?	Blood sample	28	65.12%
	Thick smear	1	2.33%
	I have not had an exam	8	18.60%
	I do not remember	6	13.95%

* Multiple choice questions.

**Table 4:** Attitudes towards malaria of the participants from Villa Grande, El Bagre.

Variable		Participants (*n* = 43)	%
Do you take or have you taken all the pills for treatment?	Yes	41	95.35%
	No	2	4.65%
Do you agree with spraying insecticides?	Yes	41	95.35%
	No	1	2.33%
	Do not know/No answer	1	2.33%
How is malaria healed? *	Going to the hospital	19	44.19%
	Taking traditional medicine	11	25.58%
	Taking the treatment formulated by the doctor	11	25.58%
	Going to the microscopist health post	4	9.30%
	Going to private doctor	2	4.65%
Do you know anyone who cures malaria using traditional medicine?	Yes	12	27.91%
	No	31	72.09%

* Multiple choice questions.

**Table 5:** Practices for malaria prevention of the participants from Villa Grande, El Bagre.

Variable		Participants (*n* = 43)	%
How do you prevent getting malaria? *	Using mosquito nets	28	65.12%
	Puncturing objects that may contain water	4	9.30%
	Draining ponds and stagnant waters	3	6.98%
	Fumigation	2	4.65%
	Personal protection (clothing)	1	2.33%
	Other		
	Using a fan	10	23.26%
	Do not drink unboiled/ dirty water	3	6.98%
	Do not bathe in streams	2	4.65%
	Burning material to generate smoke	2	4.65%

* Multiple choice questions.

**Table 6:** Association between sociodemographic determinants or malaria infection and knowledge score for participants in Villa Grande, El Bagre.

Determinants		Knowledge					
		Good *n* (%)	Poor *n* (%)	Odds Ratio	CI 95%	*X* ^2^	*p* Value
**Sociodemographic characteristics**							
Gender	Female	17 (62.96%)	10 (37.04%)	3.74	1.00–13.92	2.87	0.04 *
	Male	5 (31.25%)	11 (68.75%)				
Age	<40	13 (52.00%)	12 (48.00%)	1.08	0.32–3.64	0	0.57
	>40	9 (50.00%)	9 (50.00%)				
Education (any level)	Yes	18 (54.54%)	15 (45.56%)	1.80	0.42–7.58	0.19	0.32
	No	4 (40.00%)	6 (60.00%)				
Occupation ^§^	Homemaker	16 (64.00%)	9 (36.00%)	3.55	0.99–12.73	2.80	0.04 *
	Agriculture	3 (50.00%)	3 (50.00%)	0.94	0.16–5.31	0	0.64
	Mining	0 (0%)	3 (100%)	0	Undefined	1.53	0.10
	Other	3 (33.33%)	6 (66.66%)	0.40	0.08–1.84	0.68	0.20
**Previous malaria infection**	Yes	14 (58.33%)	10 (41.66%)	1.92	0.56–6.51	0.56	0.22
	No	8 (42.10%)	11 (57.89%)				

* *p* value < 0.05 (in bold). ^§^ Each of the occupations was compared against the others.

**Table 7:** Association between sociodemographic determinants or malaria infection and knowledge score for participants in Villa Grande, El Bagre.

Determinants		Attitude					
		Positive *n* (%)	Negative *n* (%)	Odds Ratio	CI 95%	*X* ^2^	*p* Value
**Sociodemographic characteristics**							
Gender	Female	24 (88.88%)	3 (11.11%)	4.80	0.99–23.07	2.70	0.04 *
	Male	10 (62.50%)	6 (37.50%)				
Age	<40	15 (83.33%)	3 (16.66%)	1.57	0.33–7.38	0.04	0.42
	>40	19 (76.00%)	6 (24.00%)				
Education (any level)	Yes	26 (78.78%)	7 (21.21%)	0.92	0.15–5.25	0.00	0.65
	No	8 (80.00%)	2 (20.00%)				
Occupation ^§^	Home	22 (88.00%)	3 (12.00%)	3.66	0.77–17.34	1.70	0.09
	Agriculture	4 (66.66%)	2 (33.33%)	0.45	0.07–3.07	0.06	0.36
	Mining	2 (66.66%)	1 (33.33%)	0.5	0.04–6.22	0	0.51
	Other	6 (66.66)	3 (33.33%)	0.42	0.08–2.21	0.32	0.27
**Previous malaria infection**	Yes	18 (75.00%)	6 (25.00%)	0.56	0.12–2.62	0.12	0.36
	No	16 (84.21%)	3 (15.78%)				

* *p* value < 0.05 (in bold). ^§^ Each of the occupations was compared against the others.

## Data Availability

All relevant data is presented within the manuscript.

## Supplementary Materials

The following supporting information can be downloaded at https://www.mdpi.com/article/10.3390/tropicalmed9110281/s1, Table S1: Score determined for knowledge, attitudes and practices about malaria for participants in Villa Grande, El Bagre; Table S2: Analysis of the association between sociodemographic determinants or malaria infection and practice score for participants in Villa Grande, El Bagre.

## References

[1] PAHO/WHO (2020). Actualización Epidemiológica: Situación de La Malaria En Resumen de La Situación.

[2] INS (2021). Boletín Epidemiológico Semanal 52 de 2021.

[3] INS (2022). Boletín Epidemiológico Semanal 52 de 2022.

[4] INS (2023). Boletín Epidemiológico Semana 52 de 2023.

[5] Ministerio de Salud y Protección Social Malaria. https://www.minsalud.gov.co/salud/publica/PET/Paginas/malaria.aspx.

[6] Carmona-Fonseca J. (2004). Malaria in the Colombian Regions of Uraba and Bajo Cauca, Province of Antioquia: An Overwiew to Interpret the Antimalarial Therapeutic Failure. Iatreia.

[7] Gobierno de Colombia/PAHO (2020). Plan Estratégico Nacional de Malaria 2019–2022. Equipo Funcional Nacional de Malaria.

[8] Shiff C. (2018). Integrated Approach to Malaria Control. Science.

[9] Piñeros J.G. (2010). Malaria and Social Health Determinants: A New Heuristic Framework from the Perspective of Latin American Social Medicine. Biomédica.

[10] Cardona-Arias J.A., Salas-Zapata W., Carmona-Fonseca J. (2020). Systematic Review of Qualitative Studies about Malaria in Colombia. Heliyon.

[11] WHO (2021). Global Technical Strategy for Malaria 2016–2030.

[12] Minakawa N., Dida G.O., Sonye G.O., Futami K., Kaneko S. (2008). Unforeseen Misuses of Bed Nets in Fishing Villages along Lake Victoria. Malar. J..

[13] Mulualem A.G., Fentie M. (2015). Misuse of Insecticide Treated Bed Nets in Malaria Epidemic Areas of Gende Rege Kebelle, Dire Dawa Administration, Eastern Ethiopia. J. Zool. Stud..

[14] Ladu H.I., Shuaibu U., Pulford J. (2024). Reasons for Mosquito Net Non-Use in Malaria-Endemic Countries: A Review of Qualitative Research Published between 2011 and 2021. Trop. Med. Int. Health.

[15] Gupta R., Raina S., Shora T., Jan R., Sharma R., Hussain S. (2016). A Household Survey to Assess Community Knowledge, Attitude and Practices on Malaria in a Rural Population of Northern India. J. Fam. Med. Prim. Care.

[16] Khan S.J., Usman M., Abbass Y., Hussain M., Ali M. (2010). Kap Study on Malaria. J. Med. Sci..

[17] Liheluka E.A., Massawe I.S., Chiduo M.G., Mandara C.I., Chacky F., Ndekuka L., Temba F.F., Mmbando B.P., Seth M.D., Challe D.P. (2023). Community Knowledge, Attitude, Practices and Beliefs Associated with Persistence of Malaria Transmission in North-Western and Southern Regions of Tanzania. Malar. J..

[18] Cardona-Arias J.A., Salas-Zapata W.A., Carmona-Fonseca J. (2019). Social Determination and Determinants of Malaria: A Systematic Review, 1980–2018. Rev. Panam. Salud Publica/Pan Am. J. Public Health.

[19] Rosero C.Y., Jaramillo G.I., Montenegro F.A., García C., Coral A.A. (2020). Community Perception of Malaria in a Vulnerable Municipality in the Colombian Pacific. Malar. J..

[20] Coral A. (2019). Conocimientos, Actitudes y Prácticas Comunitarias En Malaria En Bocas de Satinga, Olaya Herrera, Colombia. Master’s Thesis.

[21] Restrepo A., Duque V., Herrera N., Díaz D., Sierra C., Gómez V. (2019). Conocimientos, Prácticas y Actitudes Sobre La Malaria En El Municipio de Lloró, Chocó, Colombia. Arch. Med..

[22] Forero D.A., Chaparro P.E., Vallejo A.F., Benavides Y., Gutiérrez J.B., Arévalo-Herrera M., Herrera S. (2014). Knowledge, Attitudes and Practices of Malaria in Colombia. Malar. J..

[23] Nieto T., Méndez F., Carrasquilla G. (1999). Knowledge, Beliefs and Practices Relevant for Malaria Control in an Endemic Urban Area of the Colombian Pacific. Soc. Sci. Med..

[24] Molineros-Gallón L.F., Hernández-Carrillo M., Castro-Espinosa J., Trujillo De Cisneros E. (2018). Knowledge, Attitudes, Perceptions and Community Practices for Urban Malaria. Tumaco, Colombia. Rev. Salud Publica.

[25] Secretaría Seccional de Salud y Protección Social de Antioquia (2023). Boletín Epidemiológico de Antioquia.

[26] Padilla-Rodríguez J.C., Olivera M.J., Ahumada-Franco M.L., Paredes-Medina A.E. (2021). Malaria Risk Stratification in Colombia 2010 to 2019. PLoS ONE.

[27] Chaparro P., Padilla J., Vallejo A.F., Herrera S. (2013). Characterization of a Malaria Outbreak in Colombia in 2010. Malar. J..

[28] Hernández-Valencia J.C., Rincón D.S., Marín A., Naranjo-Díaz N., Correa M.M. (2020). Effect of Land Cover and Landscape Fragmentation on Anopheline Mosquito Abundance and Diversity in an Important Colombian Malaria Endemic Region. PLoS ONE.

[29] Naranjo-Dıaz N., Hernandez-Valencia J.C., Gomez G.F., Correa M.M. (2023). Spatial and Temporal Diversity Variation in the *Anopheles* Communities in Malaria-Endemic Regions of Colombia. Am. J. Trop. Med. Hyg..

[30] Dean A., Sullivan K., Soe M. OpenEpi: Open Source Epidemiologic Statistics for Public Health V3.01. www.OpenEpi.com.

[31] PAHO/WHO (2008). Encuesta Sobre Conocimientos, Actitudes y Prácticas (CAP): Una Herramienta Para El Abordaje Intercultural de La Malaria.

[32] CDC Epi InfoTM, a Database and Statistics Program for Public Health Professionals 2021. https://www.cdc.gov/epiinfo/index.html.

[33] Bloom B., Engelhart M., Furst E., Hill W., Krathwohl D. (1956). Taxonomy of Educational Objetives. The Classification of Education Goals. Handbook I: Cognitive Domain.

[34] Lopez A.R., Brown C.A. (2023). Knowledge, Attitudes and Practices Regarding Malaria Prevention and Control in Communities in the Eastern Region, Ghana, 2020. PLoS ONE.

[35] Masuda K. (2020). Length of Maternal Schooling and Children’s Risk of Malaria Infection: Evidence from a Natural Experiment in Uganda. BMJ Glob. Health.

[36] Rudasingwa G., Cho S. (2024). il Malaria Prevalence and Associated Population and Ecological Risk Factors among Women and Children under 5 Years in Rwanda. Heliyon.

[37] Yang D., He Y., Wu B., Deng Y., Li M., Yang Q., Huang L., Cao Y., Liu Y. (2020). Drinking Water and Sanitation Conditions Are Associated with the Risk of Malaria among Children under Five Years Old in Sub-Saharan Africa: A Logistic Regression Model Analysis of National Survey Data. J. Adv. Res..

[38] Bradley J., Rehman A.M., Schwabe C., Vargas D., Monti F., Ela C., Riloha M., Kleinschmidt I. (2013). Reduced Prevalence of Malaria Infection in Children Living in Houses with Window Screening or Closed Eaves on Bioko Island, Equatorial Guinea. PLoS ONE.

[39] Nguela R.L., Bigoga J.D., Armel T.N., Esther T., Line D., Boris N.A., Frederic T., Kazi R., Williams P., Mbacham W.F. (2020). The Effect of Improved Housing on Indoor Mosquito Density and Exposure to Malaria in the Rural Community of Minkoameyos, Centre Region of Cameroon. Malar. J..

[40] Nkuo-Akenji T., Ntonifor N.N., Ndukum M.B., Kimbi H.K., Abongwa E.L., Nkwescheu A., Anong D.N., Songmbe M., Boyo M.G., Ndamukong K.N. (2006). Environmental Factors Affecting Malaria Parasite Prevalence in Rural Bolifamba, South-West Cameroon. Afr. J. Health Sci..

[41] Wanzirah H., Tusting L.S., Arinaitwe E., Katureebe A., Maxwell K., Rek J., Bottomley C., Staedke S.G., Kamya M., Dorsey G. (2015). Mind the Gap: House Structure and the Risk of Malaria in Uganda. PLoS ONE.

[42] Griffith M., Rovira J., Torres R., Calzada J., Victoria C., Cáceres L. (2015). Conocimientos, Actitudes y Prácticas Sobre La Malaria En La Población Indígena Guna de La Comarca de Madungandí, Panamá, 2012. Biomédica.

[43] Pereira E.A., Ishikawa E.A., Fontes C.J. (2011). Adherence to *Plasmodium vivax* Malaria Treatment in the Brazilian Amazon Region. Malar. J..

[44] Gomes M., Wayling S., Pang L. (1998). Interventions to Improve the Use of Antimalarials in South-East Asia: An Overview. Bull. World Health Organ..

[45] Ministerio de Salud y Protección Social (2022). Guía de Práctica Clínica Diagnóstico y Tratamiento de La Malaria.

[46] Ilesanmi O., Afolabi A., Iyiola O. (2021). Effect of the COVID-19 Pandemic on Malaria Intervention Coverage in Nigeria: Analysis of the Premise Malaria COVID-19 Health Services Disruption Survey 2020. Popul. Med..

[47] WHO (2020). Tailoring Malaria Interventions in the COVID-19 Response Global Malaria Programme.

[48] Zawawi A., Alghanmi M., Alsaady I., Gattan H., Zakai H., Couper K. (2020). The Impact of COVID-19 Pandemic on Malaria Elimination. Parasite Epidemiol. Control.

[49] Broekhuizen H., Fehr A., Nieto-Sanchez C., Muela J., Peeters-Grietens K., Smekens T., Kalleh M., Rijndertse E., Achan J., D’Alessandro U. (2021). Costs and Barriers Faced by Households Seeking Malaria Treatment in the Upper River Region, The Gambia. Malar. J..

[50] WHO (2013). WHO Guidance Note for Estimating the Longevity of Long-Lasting Insecticidal Nets in Malaria Control September 2013.

[51] Mejía P., Teklehaimanot H.D., Tesfaye Y., Teklehaimanot A. (2013). Physical Condition of Olyset^®^ Nets after Five Years of Utilization in Rural Western Kenya. Malar. J..

[52] CDC Insecticide-Treated Bed Net. https://www.cdc.gov/malaria/malaria_worldwide/reduction/itn.html.

[53] Hewitt S.E., Farhan M., Urhaman H., Muhammad N., Kamal M., Rowland M.W. (1996). Self-Protection from Malaria Vectors in Pakistan: An Evaluation of Popular Existing Methods and Appropriate New Techniques in Afghan Refugee Communities. Ann. Trop. Med. Parasitol..

[54] Heggenhougen H.K., Hackethal V., Vivek P. (2003). The Behavioural and Social Aspects of Malaria and Its Control. An Introduction and Annotated Bibliography.

[55] Naranjo-Díaz N., Altamiranda-Saavedra M., Correa M.M. (2019). Anopheles Species Composition and Entomological Parameters in Malaria Endemic Localities of North West Colombia. Acta Trop..

[56] Diiro G.M., Affognon H.D., Muriithi B.W., Wanja S.K., Mbogo C., Mutero C. (2016). The Role of Gender on Malaria Preventive Behaviour among Rural Households in Kenya. Malar. J..

[57] Tolhurst R., Nyonator F.K. (2006). Looking within the Household: Gender Roles and Responses to Malaria in Ghana. Trans. R. Soc. Trop. Med. Hyg..

[58] Alvarado B.E., Gómez E., Serra M., Carvajal R., Carrasquilla G. (2006). Evaluación de Una Estrategia Educativa En Malaria Aplicada En Localidades Rurales Del Pacífico Colombiano. Biomédica.

