# Supplementary material for: Community Knowledge, Attitudes and Practices About Malaria: Insights from a Northwestern Colombian Endemic Locality
Source: Trop Med Infect Dis. 2024 Nov 18;9(11):281. doi: 10.3390/tropicalmed9110281 (PMC11598121; doi:10.3390/tropicalmed9110281)
Supplement: Supplementary file 1 [file tropicalmed-09-00281-s001.zip › tropicalmed-3251592-supplementary.pdf]

Table S1. Score determined for knowledge, attitudes and practices about malaria for participants in Villa Grande, El Bagre.

**Knowledge**		**Attitudes**		**Practices**	
**Knowledge level**	***n* (%)**	**Attitudes level**	***n* (%)**	**Practices level**	***n* (%)**
Good >60%	22 (51.16%)	Positive >60%	34 (79.07%)	Positive >60%	2 (4.65%)
Poor <60%	21 (48.44%)	Negative <60%	9 (20.09%)	Negative <60%*	41 (95.35%)

*Most negative practice ratings have a score of 50%.

Table S2. Association between sociodemographic determinants or malaria infection with practice score for participants in Villa Grande, El Bagre.

**Determinants**		**Practice**					
		**Positive *n* (%)**	**Negative *n* (%)**	**Odds ratio**	**CI95%**	X^2	***p* value**
**Socio-demographic characteristics**							
Gender	Female	2 (7.41%)	25 (92.59%)	0	Undefined	0.13	0.38
	Male	0 (0%)	16 (100%)				
Age	<40	1 (5.56%)	17 (94.44%)	1.41	0.08-24.17	0	0.66
	>40	1 (4.00%)	24 (96.00%)				
Education (any level)	Yes	2 (6.06%)	31 (93.4%)	0	Undefined	0	0.58
	No	0 (0%)	10 (100%)				
Occupation §	Home	2 (8.00%)	23 (92.00%)	0	Undefined	0.24	0.33
	Agriculture	0 (0%)	6 (100%)	0	Undefined	0	0.73
	Mining	0 (0%)	3 (100%)	0	Undefined	0	0.86
	Other	0 (0%)	9 (100%)	0	Undefined	0	0.62
Previous malaria infection	Yes	1 (4.17%)	23 (95.80%)	0.78	0.04-13.39	0	0.69
	No	1 (5.26%)	18 (94.74%)				

* *p* value <0.05

§ Each of the occupations was compared against the others.
